# Corn Stalk Disease*Read before the California State Veterinary Association.

**Published:** 1892-02

**Authors:** H. F. Spencer


### CORN STALK DISEASE.*

By H. F. Spencer, D. V. S.

Corn stalk disease by many was supposed to be a specific form of fardle-bound, or impaction of the omasum, and by others to be due to the animal partaking of large quanities of corn smut or ustilago maidis. In the year of 1868 this disease was quite prevalent and there was also a great amount of smut on the corn.

Prof. Gamgee, on the 26th of February 1868, commenced to feed smut to two cows, each taking one and a half pounds of corn meal, and three ounces of usilago, three times a day, one receiving her feed wet, the other dry. On the 7th of March the dose was increased to four ounces and again on the 15th of the same month it was increased to twelve ounces; the cow on dry feed lost some flesh, the other gained; the only noticeable change being that the faces were darkened. Thus in three weeks these two cows consumed forty-two pounds of pure smut, and considering the lightness of this fungus we can readily see that it would be impossible for an animal to gather that amount in the field.

In regard to impaction of the omasum, post mortems have often revealed its entire absence and where it does exist we find that it is by no means the only lesion, but that the entire viscera are more or less affected, and further that the disease never manifests itself in less than six days, while it often does not appear until fourteen days have elapsed, from the date of the animal's admission to the corn field.

The researches of Dr. Frank S. Billings instructor of micro-histology and gross pathology at the Chicago Veterinary College and also Chief of the Nebraska Experimental Station, and of Prof. Foster and Prof. Burrill, Botanist of the University at Champaigne, Ill., furnish conclusive evidence that the disease is due to a germ. In 1881 Prof. Foster discovered a parasite of the corn which was later and further investigated by Prof. Burrill. The presence of this germ in a field of corn, may be determined by patches showing a stunted appearance; the lower leaves first turn greenish-yellow, then yellow, and exhibit brownish watery looking spots and often find gelatinous like exudate on the stalks. The germ can be ob-

* Read before the California State Veterinary Association.

tained from either of these places, the parasite may be fonnd in broom corn, maize, rye, etc.

In 1889 Dr. Billings isolated the germ taken from a pathological specimen from a cow that died of corn stalk disease on a farm near Fremont, Neb., and this germ was found to be identical with the one discovered by Prof. Foster, and was recognized as such by Prof. Burrill. With pure cultures of this germ Dr. Billings inoculated rabbits and guinea pigs, which died with symptoms and presented lesions similar to those seen in cattle, that died after having been turned into corn fields.

Young cattle are more frequently attacked which might indicate that older ones had recovered from a mild type of the malady, and thereby secured for themselves immunity from a second attack. Dr. Billings describes the disease as follows: An acute extra organismal septicemia, due to a micro organism belonging to the class of ovoid belted germs, to which variety also belong the germ of swine plague, Texas fever, hen cholera and yellow fever, but in no case are the germs identical, but each a specific germ producing a specific disease.

The first evidence of the disease is a rapid elevation of temperature from 106 to 107, then either drowsiness, or delirium, if the latter the animal bellow and chase small animals as pigs, chickens, etc.; soon symptoms develope in the organ affected by the septicemia then coma, and death shortly, generally in about six hours after the appearance of the trouble, however they may die in two hours or even live two days. Horses and all other herbivora may contract this disease. Post-mortem reveals dark coffee-colored blood with slight tendency to coagulate, the red corpuscles have a shrivelled appearance, as though subjected to acetic acid. We may find conjestion of lungs, the spleen enlarged and the structure broken down, the liver is inflamed; we may have inflamation of any of the internal viscera.

Therapeutical treatment is of no avail, but if only small quantities of the germ have been introduced into the system, and the blood is not in proper condition for their development, the animal will recover the prophylactic treatment. Do not turn cattle in a field where diseased leaves can be seen, remove healthy cattle from those ailing. Cold weather does not affect the germ, therefore always burn the infected fields and all litter and excrement. All dead bodies should be cremated; quarantine fields and infected herds.

